# Serum Interleukin-26 Is a New Biomarker for Disease Activity Assessment in Systemic Lupus Erythematosus

**DOI:** 10.3389/fimmu.2021.663192

**Published:** 2021-05-14

**Authors:** Benoit Brilland, Maxime Bach-Bunner, Christopher Nunes Gomes, Vincent Larochette, Etienne Foucher, Marc Plaisance, Patrick Saulnier, Nathalie Costedoat-Chalumeau, Pascale Ghillani, Cristina Belizna, Yves Delneste, Jean-François Augusto, Pascale Jeannin

**Affiliations:** ^1^ CHU Angers, Service de Néphrologie-Dialyse-Transplantation, Angers, France; ^2^ Univ Angers, CHU Angers, INSERM, CRCINA, Angers, France; ^3^ CHU Angers, Service de Médecine interne, Angers, France; ^4^ CHU Angers, Service des Maladies du sang, Angers, France; ^5^ Service de Pharmacologie et d’Immunoanalyse, Commissariat à l’Energie Atomique Saclay, iBiTec-S, Gif sur Yvette, France; ^6^ Univ Angers, INSERM, CNRS, MINT, Angers, France; ^7^ CHU Angers, Département de Bio-Statistiques et de Méthodologie, Angers, France; ^8^ Internal Medicine Department, Referral Center for Rare Autoimmune and Systemic Diseases, Hospital Cochin, Paris, France; ^9^ Univ Angers, INSERM, CNRS, MitoVasc, Angers, France; ^10^ CHU Angers, Service d’Immunologie et Allergologie, Angers, France

**Keywords:** IL-26, SLEDAI, Proteinuria, anti-DNA antibodies, complement consumption, systemic lupus erythematosus

## Abstract

**Objective:**

Interleukin-26 (IL-26) has a unique ability to activate innate immune cells due to its binding to circulating double-stranded DNA. High levels of IL-26 have been reported in patients with chronic inflammation. We aimed to investigate IL-26 levels in patients with systemic lupus erythematosus (SLE).

**Methods:**

IL-26 serum levels were quantified by ELISA for 47 healthy controls and 109 SLE patients previously enrolled in the PLUS study. Performance of IL-26 levels and classical markers (autoantibodies or complement consumption) to identify an active SLE disease (SLE disease activity index (SLEDAI) score > 4) were compared.

**Results:**

IL-26 levels were significantly higher in SLE patients than in controls (4.04 ± 11.66 and 0.74 ± 2.02 ng/mL; p = 0.005). IL-26 levels were also significantly higher in patients with active disease than those with inactive disease (33.08 ± 21.06 vs 1.10 ± 3.80 ng/mL, p < 0.0001). IL-26 levels correlated with SLEDAI score and the urine protein to creatinine ratio (uPCR) (p < 0.001). Patients with high IL-26 levels had higher SLEDAI score, anti-DNA antibodies levels, and uPCR (p < 0.05). They presented more frequently with C3 or C4 complement consumption. Lastly, IL-26 showed stronger performance than classical markers (complement consumption or autoantibodies) for active disease identification.

**Conclusions:**

Our results suggest that, in addition to classical SLE serological markers, the measurement of IL-26 levels may be a useful biomarker for active disease identification in SLE patients.

## Introduction

Systemic lupus erythematosus (SLE) is a systemic autoimmune disease associated with the presence of autoantibodies and the formation of immune complexes that cause tissue damage (mainly targeting the skin, kidneys, and brain). Autoantibodies directed against double-stranded (ds) DNA play a key role in the initiation and maintenance of inflammatory lesions in SLE (1). Such autoantibodies were the first identified DNA shuttling molecules allowing extracellular DNA (*i.e.* released by dying cells) to be internalized *via* the FcγR and processed by antigen-presenting cells (APCs). The activation of APCs, which is required to initiate antigen-specific immune responses, is then induced *via* intracellular DNA sensors, including TLR9 (2). This process induces the production of type I interferons (IFN-I) by plasmacytoid dendritic cells (pDCs), which play a central role in the pathogenesis of lupus (3). In addition, SLE patients have elevated levels of circulating endogenous nucleic acids and of TLR9 in circulating mononuclear cells, supporting the hypothesis of a pathological role for anti-DNA autoantibodies (4). A similar DNA-shuttling activity was next reported for members of the cathelicidin family, especially LL-37. Cathelicidins are cationic and amphipathic molecules that exhibit antimicrobial activity. LL-37-mediated internalization of extracellular DNA induces the activation of several signaling pathways (TLR, STING, inflammasome) (5–7). Its ability to render extracellular DNA inflammatory has been demonstrated in several autoimmune diseases, including SLE (8). Nevertheless, anti-DNA Abs and anti-microbial peptides are not always found in SLE patients, suggesting the existence of other DNA shuttling molecules.

IL-26 was initially described as a molecule overexpressed by CD8^+^ T lymphocytes transformed by a Herpesvirus (9). This cytokine was classified in the IL-20 cytokine sub-family (10). IL-26 is an amphipathic and cationic protein (calculated pHi = 10.7) secreted as a 36 kDa homodimer (11) that preferentially interacts with glycosaminoglycans expressed on cell surfaces (9, 11, 12). IL-26 was initially identified as a pro-inflammatory cytokine (11, 13), inducing the production of inflammatory cytokines by myeloid cells that are involved in the differentiation of naive CD4^+^ T cells into Th17 cells (14). Th17 cells themselves are also a major source of IL-26 (13, 14), leading to an inflammatory amplification loop. Importantly, we and others have reported that, due to its biochemical characteristics, IL-26 binds to extracellular DNA and favors its internalization by monocytes and pDCs, leading to the production of IFN-I and inflammatory cytokines (2, 14–16).

The involvement of IL-26 in numerous chronic auto-inflammatory diseases (2, 13–15, 17–19) and in two major pathogenic processes in SLE, namely the induction of IFN-I and its production by Th17 lymphocytes, makes the analysis of its role in SLE essential. We report that the levels of IL-26 are higher in SLE patients than healthy subjects and show a significant correlation with disease activity. Thus, in addition to classical serological markers, IL-26 levels may help in SLE activity assessment.

## Methods

### Study Design and Clinical Diagnosis of SLE

This study is a post-hoc analysis of the PLUS study (20), a prospective randomized, double-blind, placebo-controlled, multicenter study aiming to compare standard and adjusted hydroxychloroquine (HCQ) dosing schedules to reduce SLE flares. It was conducted from June 2007 through August 2010 in 37 French centers. Adults with a diagnosis of SLE, according to the 1997-revised American College of Rheumatology (ACR) classification criteria (21), from at least 6 months, were included. Patients had to be under stable treatment (HCQ, steroids, or an immunosuppressive regimen, such as cyclophosphamide, methotrexate, or mycophenolic acid) for at least 2 months, and to have a Safety of Estrogens in Lupus Erythematosus National Assessment SLE Disease Activity Index (SELENA-SLEDAI) ≤ 12. The main exclusion criteria were an estimated glomerular filtration rate (eGFR, calculated from serum creatinine according to the Cockcroft–Gault equation) < 60 mL/min/1.73 m^2^, chronic alcoholism, and liver failure. This study followed the recommendations of the Helsinki Declaration and all participants provided written informed consent to participate.

### Data Analysis

Data concerning the age, sex, SLE criteria, years since SLE diagnosis, anti-phospholipid syndrome (APS) criteria (with both clinical and laboratory criteria, as defined by the 2006 International consensus criteria (22)), SLEDAI score, creatinine, eGFR, urine protein to creatinine ratio (uPCR), antinuclear Ab titers assessed in a centralized manner by indirect immunofluorescence (IIF) analysis on Hep2 cells (HEp2000 slides, ImmunoConcepts, positivity set at 1/80), anti-dsDNA (Farr assay, Trinity Biotech and Eti-dsDNA, DiaSorin), C3 and C4 complement levels were collected for each SLE patient. Patients with a SLEDAI ≤ 4 were considered to be in an inactive disease state, whereas those with a SLEDAI > 4 were considered to be in an active disease state.

### IL-26 Quantification

IL-26 was quantified in serum samples collected between 2007 and 2009 for the PLUS study. Serums were stored in -80°C. IL-26 was quantified by ELISA using home-made anti-IL-26 monoclonal antibodies (mAbs), as previously described (15, 16). Sensitivity and specificity of this assay are detailed in **Supplementary Figure 1**. The detection limit is 200 pg/ml. Briefly, 96-well plates (Maxisorp; Nunc) were coated with 5 µg/mL anti-IL-26 mAb (clone 13C9). After saturation with phosphate-buffered saline containing 5% bovine serum albumin (w/v), plates were successively incubated with the serum samples, 1 µg/mL biotinylated anti-IL-26 mAb (clone 8G3), and then with streptavidin-horseradish peroxidase (BD Biosciences). Bound Abs were detected using the TMB substrate (Sigma-Aldrich). The optical density was measured at λ = 450 nm. IL-26 was also quantified in serum samples from 47 healthy human volunteers (Blood Collection Center, Angers, France; agreement PLER ANG 2017-01). As IL-26 can bind to circulating DNA we made sure that the latter did not interfere with IL-26 detection (**Supplementary Figure 1C**).

### Statistical Analysis

Subject characteristics are reported as numbers and percentages for categorical variables and as the mean ± standard deviation or median [Inter-Quartile Range (IQR)], when appropriate, for continuous variables. Data were compared using the Chi² test for categorical variables or Student t-test for continuous variables. For non-normal continuous variables, Mann-Whitney or Kruskal-Wallis tests (followed by Dunn post-hoc test for multiples comparisons when applicable) were used. Spearman correlation tests were used to evaluate the link between IL-26 and other parameters. Performances of markers for active SLE identification were compared using McNemar test (for sensitivities and specificities) (23) or Generalized Score Statistic (for negative and positive predictive values) (24). Statistical analyses were performed using R software version 3.6. P-values < 0.05 were considered significant.

## Results

### Patients Characteristics

Among 109 patients included in this study, 94 were female (86.2%) and the mean age was 41.1 ± 9.8 years. SLE had been diagnosed an average of 12.9 ± 7.3 years before inclusion in the PLUS study. The mean SLEDAI score was 1.6 ± 2.2. As expected, given the exclusion criteria, the eGFR was > 60 mL/min/1.73 m². These data are summarized in **Table 1**. Characteristics of healthy subjects (age, sex) were not available.

**Table 1:** SLE patient characteristics.

	All cohort, N = 109	IL-26 “low”, N = 88 (81%)	IL-26 “high”, N = 21 (19%)	p-value
**Baseline characteristics**				
Age	38 [33-45]	38 [33-46]	39 [33-44]	0.6
Female sex	94 (86%)	78 (89%)	16 (76%)	0.2
Years since SLE diagnosis	8 [5-14]	7 [5-13]	11 [10-14]	**0.036**
SLEDAI	1 [0-2]	0 [0-2]	4 [4-6]	**< 0.001**
SLEDAI > 4	10 (9.2%)	0 (0%)	10 (48%)	**< 0.001**
**Biological presentation**				
Antiphospholipid syndrome	55 (50%)	42 (48%)	13 (62%)	0.4
Antinuclear antibodies titer	640 [160-1280]	640 [160-1280]	640 [320-1280]	0.16
Antinuclear antibodies titer (ranges)				0.09
0 - 1/200	29 (27%)	27 (31%)	2 (9.5%)	
1/200 - 1/500	20 (18%)	14 (16%)	6 (29%)	
> 1/500	60 (55%)	47 (53%)	13 (62%)	
Anti-DNA				
Farr assay (UI/mL)	6 [5-16]	5 [5-10]	17 [9-53]	**< 0.001**
Positivity in Farr assay (> 9 UI/mL)	38 (35%)	23 (26%)	15 (71%)	**< 0.001**
ELISA (UI/mL)	18 [7-52]	15 [6-52]	27 [20-55]	**0.035**
Positivity in ELISA (> 28 UI/mL)	42 (39%)	32 (36%)	10 (48%)	0.5
Urine Protein to Creatinine ratio				
uPCR (g/g)	0.0 [0.0-0.1]	0.0 [0.0-0.1]	0.1 [0.0-0.3]	**0.003**
Significant uPCR (> 0.5 g/g)	5 (4.6%)	1 (1.1%)	4 (19%)	0.7
eGFR (mL/min/1.73 m²)	108 [86-131]	103 [84-130]	119 [96-133]	0.3
Creatininemia (µmol/L)	65 [58-73]	65 [58-72]	66 [61-80]	0.5
Complement components				
C3 (g/L)	1.02 [0.88-1.15]	1.03 [0.91-1.16]	0.88 [0.68-1.05]	**0.013**
Low C3 levels (< 0.7 g/L)	8 (7.3%)	2 (2.3%)	6 (29%)	**< 0.001**
C4 (g/L)	0.19 [0.15-0.24]	0.19 [0.16-0.24]	0.16 [0.11-0.24]	0.2
Low C4 levels (< 0.15 g/L)	26 (24%)	17 (19%)	9 (43%)	**0.047**
IL-26 level (ng/ml)	0 [0-0]	0 [0-0]	12 [6-28]	**< 0.001**
**Ongoing therapy**				0.12
None	39 (36%)	34 (39%)	5 (24%)	
Steroids or immunosuppressive therapy	54 (50%)	44 (50%)	10 (48%)	
Both	16 (15%)	10 (11%)	6 (29%)	

Data are shown as median [interquartile range] for continuous variables and as number (%) for categorical variables.

eGFR, estimated glomerular filtration rate; SLE, systemic lupus erythematosus; SLEDAI, SLE disease activity; uPCR, urine protein to creatinine ratio. Significant p-values are in bold.

### IL-26 Levels Were Higher in Patients With SLE

**Figure 1:**
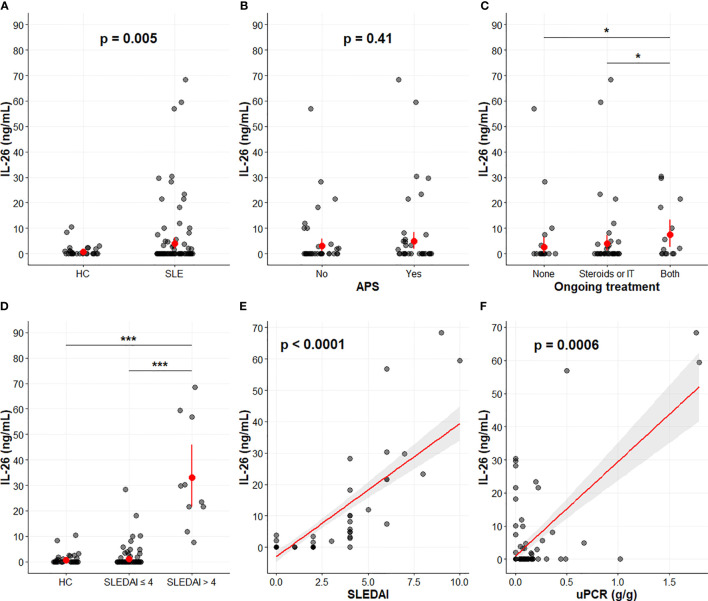
IL-26 levels in SLE. IL-26 levels according to SLE status **(A)**, APS status **(B)**, and ongoing treatment **(C)**. Correlation between IL-26 levels and SLEDAI **(D, E)** or the urine protein to creatinine ratio **(F)**. **(A–D)** In red, mean ± 95% confidence interval. **(E, F)** Graphs depict the linear regressions (red) with the 95% confidence intervals (grey). Spearman correlation coefficients (ρ) were 0.70 (p < 0.0001) between IL-26 and SLEDAI **(E)** and 0.32 (p = 0.0006) between IL-26 and uPCR **(F)**. *p < 0.05. ***p < 0.001. APS, antiphospholipid syndrome; HC, healthy controls; IT, immunosuppressive therapy; SLE, systemic lupus erythematosus; SLEDAI, systemic lupus erythematosus disease activity index; uPCR, urine protein to creatinine ratio.

The levels of serum IL-26 were significantly higher in SLE patients than in healthy subjects (4.04 ± 11.66 and 0.74 ± 2.02 ng/mL, respectively; p = 0.005) (**Figure 1A**). No difference in IL-26 levels was observed according to the APS status (p = 0.41) (**Figure 1B**). IL-26 levels were higher in patients undergoing both steroids and immunosuppressive treatment (p = 0.01) (**Figure 1C**).

### IL-26 Levels Correlated With Disease Activity (SLEDAI) and Proteinuria

**Figure 2:**
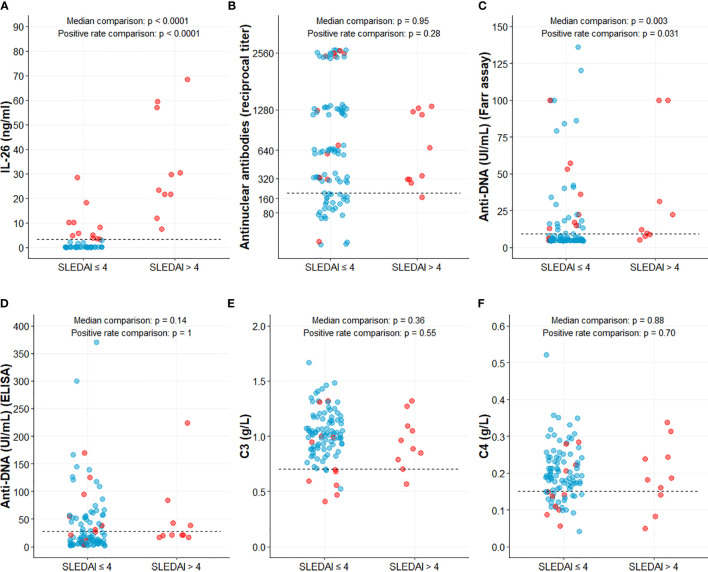
IL-26 and classical markers according to SLE disease activity. IL-26 **(A)**, Antinuclear antibodies **(B)**, Anti-DNA antibodies **(C, D)**, C3 **(D)** and C4 **(E)** complement components levels. Data were compared with Mann-Whitney test (for median comparison) and with Fisher exact test [for percentage of positive patients (above threshold)]. Dashed line represents positivity threshold, set at 3.2 ng/ml for IL-26 **(A)**, 1/200 for antinuclear antibodies **(B)**, 9 and 28 UI/mL for anti-DNA antibodies (Farr assay and ELISA, C and D, respectively) or lower normal value (0.7 and 0.15 g/L) for C3 and C4 complement component respectively **(E, F)**. Blue and red dots represent patients in the low or high IL-26 groups respectively.

We examined the potential relationship between SLE disease activity and IL-26 expression by comparing the IL-26 levels in patients with high (active disease, n = 10) or low SLEDAI score (inactive disease, n = 99). IL-26 levels were significantly higher in patients with active disease than patients with inactive disease (33.08 ± 21.06 versus 1.10 ± 3.80 ng/mL, respectively; p < 0.0001) (**Figures 1D** and **2A**).

IL-26 levels strongly correlated with the SLEDAI (ρ = 0.70, p < 0.0001) (**Figure 1E**) and also, to a lower extent, with uPCR (ρ = 0.32, p = 0.0006) (**Figure 1F**). No correlation between IL-26 levels and creatininemia or eGFR, nor with autoantibody titers, was observed (data not shown).

### Patients With High IL-26 Levels Had a Higher Clinical and Serological Active Disease

Patients with IL-26 levels higher than 3.2 ng/mL (best threshold according to area under ROC curve for SLE diagnosis) were further included in the “high IL-26 levels” (IL-26^high^) group. Twenty-one SLE patients (19.3%) and 2 (4.3%) healthy controls (p = 0.015) had “high” IL-26 levels. IL-26^high^ patients had a disease that was significantly diagnosed longer ago (p = 0.036) and had a significantly higher SLEDAI score than IL-26^low^ patients (p < 0.001). In IL-26^high^ patients, C3 or C4 complement component were significantly more frequently lowered (p < 0.001 and p = 0.047 respectively). Anti-DNA antibodies levels (p = 0.001) and proteinuria were significantly higher (p = 0.003), while antinuclear antibodies tended to be more frequently found at higher levels (p = 0.09). None of the IL-26^low^ patients had an active disease (SLEDAI ≤ 4) (**Table 1**).

### IL-26 Showed Stronger Performance Than Classical Markers for Active Disease Identification

Unlike classical markers (antinuclear antibodies higher than 1/200, anti-DNA antibodies, C3 and C4 levels), which were found abnormal in only a fraction of patients with an active disease (90%, 40-70%, 10% and 30%, respectively), IL-26 levels were elevated in all (100%) of them (**Figure 2**). Thus, elevated IL-26 levels had the best performance among the classic markers for active SLE identification. Indeed, a high IL-26 level identified active SLE with similar or, in most cases, greater sensitivity, specificity, positive and negative predictive values when compared to autoantibodies (antinuclear antibodies above 1/200, anti-DNA positivity) or complement consumption (**Table 2**).

**Table 2:** Classical markers and IL-26 performance for active SLE identification (SLEDAI > 4).

	Sensitivity	Specificity	PPV	NPV
Anti-nuclear antibodies (≥ 1/200)	90% [55-100]*	28% [20-38] ***	11% [05-20] ***	97% [82-100] *
Anti-DNA antibodies (Farr) (positivity)	70% [35-93] **	69% [59-78] ***	18% [08-34] ***	96% [88-99] **
Anti-DNA antibodies (ELISA) (positivity)	40% [12-74] **	62% [51-71] ***	10% [03-23] ***	91% [82-97] **
C3 levels (below normal range)	10% [00-45] **	93% [86-97] ***	12% [00-53] ***	91% [84-96] **
C4 levels (below normal range)	30% [07-65] **	77% [67-85] ***	12% [02-30] ***	92% [83-97] **
IL-26 (above “high” threshold)	100% [69-100] **	89% [81-94] ***	48% [26-70] ***	100% [96-100] **

P-values of the comparison of sensitivities, specificities, PPV and NPV for each classical marker (versus IL-26) are depicted by stars (* < 0.05, ** < 0.01, *** < 0.001).

PPV, positive predictive value; NPV, negative predictive value.

## Discussion

Here, we report that the levels of IL-26 are higher in SLE patients than in healthy subjects and that patients with active lupus have higher IL-26 levels than those with inactive disease. We found a significant correlation between IL-26 levels and disease activity (mainly with SLEDAI but also, interestingly, with uPCR despite the absence of impaired renal function, as defined in the exclusion criteria). Patients with high IL-26 levels exhibits the most active disease, both clinically and serologically. Our results underline a strong performance for IL-26 in active SLE identification in a population with known SLE and an overall low SLEDAI score.

During sterile chronic inflammation, extracellular self-DNA, probably released during episodes of massive cell death, is a major pro-inflammatory stimulus, especially in SLE patients (25). Indeed, sequestered into organites (nucleus and mitochondria) under physiological conditions, DNA can be released into the extracellular environment following accidental cell death (necrosis) or when the clearance of apoptotic cells is delayed or impaired (leading to late apoptosis/secondary necrosis). Despite the fact that extracellular DNA is recognized as a danger signal, it does not activate immune cells, mainly because DNA sensors are sequestered intracellularly and that extracellular DNA can be degraded by nucleases, unless associated with shuttling molecules allowing it to cross membranes and access intracellular DNA sensors.

IL-26 has recently emerged as a DNA-shuttling molecule involved in the pathophysiology of chronic inflammatory disorders as well as in various infectious diseases, both viral and bacterial. We report here that SLE patients, especially those with an active disease, have elevated IL-26 levels. As shown by our group and others (2, 15), IL-26 associates with circulating nucleic acids, protects it from nucleases, and allows their internalization into cells, where they can then interact with DNA sensors, leading to the production of IFN-I and inflammatory cytokines. This process may explain the correlation between IL-26 levels and disease activity.

Our results are in agreement with data from the literature showing that IL-26 is overexpressed in chronic inflammatory diseases. IL-26 is elevated in patients with chronic rheumatic diseases, such as rheumatoid arthritis (14), spondylarthropathies (26), or vasculitis (15), as well as Th17-mediated inflammatory skin diseases, such as psoriasis (2, 18) and atopic dermatitis (27). Nevertheless, and contradictory to our results, a previous study reported that IL-26 levels were similar in SLE patients and in healthy subjects (28). This difference can be explained by differences in the methodologies used in these two studies. First, the SLE phenotype is different in Asian and Caucasian people due to differences in genetic susceptibility (29). Second, no information was provided on the IL-26 quantification assay used in the study by Zhang et al. This point is crucial as IL-26 quantification is rendered difficult due to its biochemical characteristics. Indeed, its high isoelectric point means that, in physiological conditions, IL-26 is positively charged and may easily bind to negatively charged surfaces, such as plastic. In this view, ELISA, including commercially assays, may underestimate and even give false negative results in fluids containing low levels of IL-26 (personal observations).

In the follow-up and monitoring of lupus patients, there are few biological parameters of interest, namely anti-dsDNA Ab (and/or antinuclear Ab) levels, complement activity and proteinuria (without considering interferon, which is currently not routinely assayed). All of them arise late during SLE flares. In our study, and as reported in previous studies (14, 15), there is an overlap in IL-26 levels between patients with active and inactive disease (especially for 2 patients with an inactive disease that had high IL-26 levels). Thus, further studies on a larger cohort of clinically defined SLE patients are needed before establishing a clinically significant reference range of IL-26 levels allowing discriminating patients with inactive versus active disease. Nonetheless, our findings suggest that, in addition to standard serological assays, IL-26 may be a useful marker to monitor disease activity, even in already known SLE patients under treatment for whom classical serological markers would fail to identify an active disease. Indeed, IL-26 showed excellent sensitivity and specificity for identifying patients with an active disease, thus surpassing the classical serological markers in this purpose. Lastly, the correlation between IL-26 levels and the uPCR, even though patients with severe renal impairment were excluded, suggests that IL-26 could be useful to monitor the risk of upcoming renal injury and should be carefully investigated in further studies.

Our study had several limitations. First, given that patients with an eGFR < 60 mL/min/1.73 m^2^ were excluded and no longitudinal follow-up was available, we cannot draw firm conclusions concerning the risk of developing lupus nephritis. Moreover, we did not monitor IL-26 levels over time which did not allow us to evaluate its potential to predict flare. Lastly, we must keep in mind that, as C-reactive protein, erythrocyte sedimentation rate or other pro-inflammatory cytokines, IL-26 appears as a non-specific marker of inflammation, since elevated levels have been reported in several inflammatory states, whether autoimmune or infectious.

In conclusion, our findings show that SLE patients have elevated IL-26 levels that correlate with the SLEDAI score and that patients with high IL-26 levels had a higher clinical and serological active disease. Our results need to be validated in a larger prospective cohort to confirm its usefulness as an additional diagnostic tool to monitor disease activity, help guide treatment, and better assist the clinician in the management of SLE patients.

## Data Availability Statement

The raw data supporting the conclusions of this article will be made available by the authors, without undue reservation.

## Ethics Statement

The studies involving human participants were reviewed and approved by Comité de Protection des Personnes St Louis Hospital, Paris. The patients/participants provided their written informed consent to participate in this study.

## The PLUS Group

Leonardo Astudillo, Cristina Belizna, Nadia Belmatoug, Olivier Benveniste, Audrey Benyamine, Holly Bezanahary, Patrick Blanco, Olivier Bletry, Bahram Bodaghi, Pierre Bourgeois, Benoît Brihaye, Emmanuel Chatelus, Nathalie Costedoat-Chalumeau, Richard Damade, Eric Daugas, Christian de-Gennes, Jean-François Delfraissy, Céline Delluc, Aurélien Delluc, Pierre Duhaut, Alain Dupuy, Isabelle Durieu, Hang-Korng EA, Dominique Farge, Christian Funck-Brentano, Frédérique Gandjbakhch, Justine Gellen-Dautremer, Pascale Ghillani-Dalbin, Bertrand Godeau, Cécile Goujard, Catherine Grandpeix, Claire Grange, Lamiae Grimaldi, Gaëlle Guettrot-Imbert, Loïc Guillevin, Eric Hachulla, Jean-Robert Harle, Julien Haroche, Pierre Hausfater, Jean Jouquan, Gilles Kaplanski, Homa Keshtmand, Mehdi Khellaf, Olivier Lambotte, David Launay, Hervé Levesque, Olivier Lidove, Eric Liozon, Kim LY, Matthieu Mahevas, Kubéraka Mariampillai, Xavier Mariette, Alexis Mathian, Karin Mazodier, Marc Michel, Nathalie Morel, Luc Mouthon, Rokiya Ngack, Jacques Ninet, Eric Oksenhendler, Jean-Luc Pellegrin, Olivier Peyr, Anne-Marie Piette, Vincent Poindron, Fabienne Roux, David Saadoun, Sabrinel Sahali, Bernadette Saint-Marcoux, Françoise Sarrot-Reynauld, Yoland Schoindre, Jérémie Sellam, Damien Sene, Jacques Serratrice, Pascal Seve, Jean Sibilia, Claude Simon, Christelle Sordet, Benjamin Terrier, Salim Trad, Jean-François Viallard, Elisabeth Vidal, Bertrand Wechsler, Pierre-Jean Weiller, Noël Zahr.

## Author Contributions

CB, YD, J-FA, and PJ designed the study. BB, CN, VL, and EF carried out the experiments. BB, MB-B, and PS analyzed the data. BB and MB-B drafted the manuscript. MP, PG, and NC-C contributed reagents/analytic tools. YD, J-FA, and PJ revised the manuscript. All authors contributed to the article and approved the submitted version.

## Funding

This study was supported by institutional grants from Inserm and the University of Angers and by a grant from the University Hospital of Angers (Project Lupix). The funders had no role in study design, data collection and analysis, decision to publish, or preparation of the manuscript. This work was realized in the context of the Laboratory of excellence Immunotherapy Graft-Oncology (LabEx IGO) program supported by the National Research Agency (ANR) via the investments for the future program ANR-11-LABEX-0016-01 and by the University of Angers - University Hospital of Angers joint program (3I-Impact).

## Conflict of Interest

The authors declare that the research was conducted in the absence of any commercial or financial relationships that could be construed as a potential conflict of interest.

## Supplementary Material

Supplementary Figure 1 IL-26 ELISA properties. Representative standard curve of the home-made IL-26 ELISA **(A)**, starting from 10 ng/ml and a subsequent 2-fold dilution. Specificity of the home-made IL-26 ELISA **(B)** obtained with cytokines belonging to the IL-10 family (IL-10, IL-19, IL-20, IL-22, IL-24, IL-26, and IL-29), and tested independently at two concentrations (5 and 50 ng/ml) (mean ± SEM, n = 2 independent experiment). Pre-incubating IL-26 with various concentration of partially digested DNA did not interfere with IL-26 detection **(C)** (mean ± SEM, n = 3 independent experiments).
