# Supplementary figures and images for: Serum Interleukin-26 Is a New Biomarker for Disease Activity Assessment in Systemic Lupus Erythematosus
Source: Front Immunol. 2021 May 14;12:663192. doi: 10.3389/fimmu.2021.663192 (PMC8160525; doi:10.3389/fimmu.2021.663192)

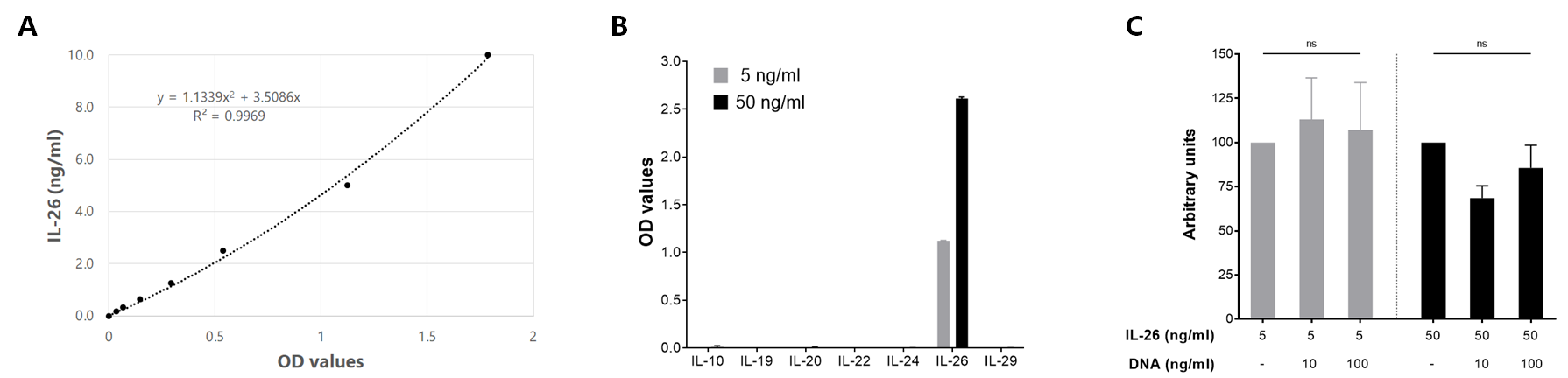

Supplement: Supplementary Figure 1 — IL-26 ELISA properties. Representative standard curve of the home-made IL-26 ELISA (A), starting from 10 ng/ml and a subsequent 2-fold dilution. Specificity of the home-made IL-26 ELISA (B) obtained with cytokines belonging to the IL-10 family (IL-10, IL-19, IL-20, IL-22, IL-24, IL-26, and IL-29), and tested independently at two concentrations (5 and 50 ng/ml) (mean ± SEM, n = 2 independent experiment). Pre-incubating IL-26 with various concentration of partially digested DNA did not interfere with IL-26 detection (C) (mean ± SEM, n = 3 independent experiments). [file Image_1.png]
